# The effects of social and foraging experiences across cognitive domains in *Polistes fuscatus*

**DOI:** 10.1016/j.anbehav.2026.123561

**Published:** 2026-04-16

**Authors:** Juanita Pardo-Sanchez, An Na Vi, Fiona Corcoran, William Kimber, Joseph A. Caldwell, Elizabeth A. Tibbetts

**Affiliations:** Ecology and Evolutionary Biology, University of Michigan, Ann Arbor, MI, U.S.A.

**Keywords:** cognition, development, early experience, ecological intelligence, foraging, plasticity, social environment

## Abstract

Experiences during development have major effects on cognitive performance. Thus far, most cognitive development research has tested how a single environmental variable influences one or a few aspects of cognition in the laboratory. Understanding cognitive variation in wild animals requires testing how performance on diverse cognitive tasks is affected by multiple interacting environmental variables in the wild. Here, we experimentally altered social and foraging experience during early adulthood in *Polistes fuscatus* paper wasps in the field. Previous work has shown that individual face learning mediates *P. fuscatus* social interactions and social experience influences how individual face learning develops. Wasps were placed in one of three treatment groups: social with foraging experience, social without foraging experience or social isolation with no foraging experience. After 10 days, we tested their individual face learning, colour learning, colour reversal and multiaccess box performance. We found that foraging experience influenced performance on the multiaccess box, as wasps with social and foraging experience opened more doors in the multiaccess box than wasps with only social experience. Foraging experience did not influence performance on other cognitive tasks, indicating that different aspects of cognition develop independently. Social experience did not influence performance on any cognitive test, a surprising result given that previous work in laboratory-raised wasps showed that social experience influences the development of individual face learning. Overall, this experiment shows different aspects of cognition develop independently of each other rather than remaining linked across rearing environments in an overall general intelligence factor. In addition, social and foraging experience during early adulthood have different effects on cognitive development.

Cognition is the processes involved in acquiring, processing and storing information, encompassing abilities such as learning, memory, problem solving and decision making (Shettleworth, 2009). Cognitive performance is thought to have major effects on how organisms interact with their environment to find food, recognize group members or choose mates (Buchanan et al., 2013). As a result, understanding the factors that influence variation in cognitive performance is a major focus of research (Deary et al., 2010; Dukas, 2004; Healy & Braithwaite, 2000). One challenge of understanding natural variation in cognitive performance is that organisms develop a range of different cognitive phenotypes in response to their biotic and abiotic environment. Most studies of cognitive development have been laboratory experiments that test how a single environmental variable influences one or a few aspects of cognition in the laboratory. However, understanding cognitive variation in wild animals requires testing how multiple interacting environmental variables impact performance on diverse cognitive tasks.

We currently have limited information about two main aspects of cognitive development in wild animals: (1) how the social and physical environment interact to influence cognitive development and (2) how environmental variation influences the development of multiple aspects of cognition. Many studies have separately tested the impact of social or physical experience on cognition (Lambert & Guillette, 2021; Rosenzweig, 2003), but much less is known about how social and physical experience interact to influence cognitive ability. Natural environments are composed of many interacting factors that may influence cognition in ways that differ from the independent effects of altering one aspect of the environment. Cognitive development research often focuses on how the environment influences a single cognitive domain such as associative learning or spatial learning (Carbia & Brown, 2019; Fischer et al., 2015; Liedtke & Schneider, 2017; van Horik et al., 2019). However, cognition is made up of multiple domains (e.g. learning, memory, behavioural flexibility) (Rowe & Healy, 2014; Shaw & Schmelz, 2017; Shettleworth, 2012). The environment may have similar effects on different aspects of cognition such that they remain linked in an overall ‘general intelligence factor’ across environments. Alternatively, the environment may influence aspects of cognition separately, such that performance on some tasks is more strongly influenced by environmental variation than is performance on other tasks. In this study, we address both gaps by testing how the social and physical environment, through long-distance foraging, interact to influence the development of multiple aspects of cognition in the wild.

Thus far, a few studies in laboratory rats have tested how the social and physical environment during early life interact to influence multiple aspects of cognition, suggesting that each influence cognition in different ways (Brenes et al., 2016; Schrijver et al., 2004; M. Z. Wang et al., 2017). Physical experience, such as larger cages or enrichment toys, improved performance on spatial cognitive tasks, such as the Morris water maze, but did not influence behavioural flexibility (Schrijver et al., 2004). In contrast, social experience increased behavioural flexibility, but not spatial cognition (Schrijver et al., 2004). Another study in rats suggests that the combination of social and physical experience may have interactive effects on rat cognition (Brenes et al., 2016). Brenes et al. (2016) used a factorial design to test how physical enrichment and/or social enrichment influenced rat performance. They found that rats with both social and physical enrichment tended to outperform rats with only physical enrichment in habituation learning and place—object recognition. The difference was not statistically significant given the small sample size, but the authors hypothesized that social and physical enrichment may have a minor additive effect on cognitive performance compared with physical enrichment alone.

While research in rats suggests that social and physical experience may influence different aspects of cognition in different ways (Brenes et al., 2016; Schrijver et al., 2004; M. Z. Wang et al., 2017), much less is known about cognitive development in nonmodel systems or in natural environments (Shaw & Schmelz, 2017). The few existing studies suggest that social and physical experience may have different effects on performance (Ashton et al., 2018; Dalesman & Lukowiak, 2011; Dickel et al., 2000; Liedtke & Schneider, 2017; Shaw et al., 2015). For example, social housing improved how accurately jumping spiders learned to associate colour with food, while physical enrichment had no effect on colour learning (Liedtke & Schneider, 2017). Both physical and social experience increased cuttlefish memory for how to extract a food reward from a tube, although the effects differed across the cuttlefish life span. Social isolation had the most detrimental effect late in life, while lack of physical enrichment had similar effects across the life span (Dickel et al., 2000). To better understand cognitive development, we need additional data on the development of multiple aspects of cognition in nonmodel systems.

*Polistes fuscatus* are a good system to test how the development of multiple aspects of cognition are influenced by social and physical experience during early adulthood because their behaviour involves social and ecological challenges. *Polistes fuscatus* wasps are central place foragers, meaning they return to their nest after long-distance foraging trips (Dew & Michener, 1978). This presents a physical challenge to wasps because wasps must learn and remember how to navigate over long distances. *Polistes fuscatus* wasps also encounter social challenges throughout their lives because they live in social groups with individually differentiated social relationships. Wasps use individual recognition to identify individual conspecifics and manage social relationships both on and off nests (Sheehan & Tibbetts, 2009; Tibbetts, 2002; Tibbetts et al., 2020). Previous work shows that *P. fuscatus* learning is affected by experience such that the capacity for individual face learning increases with social experience (Collard et al., 2024; Pardo-Sanchez et al., 2022). Wasps with no social experience cannot individually learn faces, wasps with some levels of experience (e.g. seeing pictures of other wasps or themselves in a mirror) have an intermediate capacity for individual face learning, and socially reared wasps have the highest capacity for individual face learning (Pardo-Sanchez et al., 2022). We know less about how long-distance foraging experience affects cognition in *P. fuscatus*, but work in honeybees has found that differences in foraging experience can impact cognitive performance (Cabirol et al., 2017; 2018; Carr-Markell & Robinson, 2014). This study focuses on how experience during early adulthood influence cognitive performance, although experience at any phase of development, including the larval and pupal stage, may influence insect behaviour and cognition (Hunt, 2007; Jandt et al., 2015).

In this study, we compared cognitive performance in groups of *P. fuscatus* with varying levels of foraging and social experience in early adulthood to test how experience influences the development of multiple aspects of cognition. We raised wasps in the field and manipulated their foraging experience, social experience, or both, in three rearing environments: on the nest without long-distance foraging, on the nest with long-distance foraging, or in social isolation. Then, we measured performance on four cognitive tasks: individual face learning (a social task), colour learning (associative learning), colour reversal (behavioural flexibility) and a multiaccess box task (behavioural flexibility, innovation and inhibitory control) (Benson-Amram et al., 2016; Corcoran & Tibbetts, 2026). We also measured the exploratory behaviour of each wasp to test whether exploration varied across rearing groups. We predicted that social and long-distance foraging experience would have different effects on cognitive performance. We also predicted that rearing environment would influence different aspects of cognition in different ways such that performance on some tasks would be more strongly influenced by environmental variation than performance on other tasks.

## METHODS

### Wasp Rearing

We reared *P. fuscatus* wasps in nestboxes at the Matthaei Botanical Gardens in Ann Arbor, Michigan, U.S.A. In early June, we marked the foundresses from each nest with nontoxic model train paint. Nests were checked multiple times a week for newly eclosed individuals. Newly eclosed individuals were assigned to one of the three treatment groups (foraging, nonforaging, social isolation), each of which is described in more detail below. Nests were usually not checked over the weekend, so unmarked wasps found on Monday morning could have up to 72 h of experience. These individuals remained on their nest and were assigned to the social foraging treatment group to ensure their experience did not influence experimental results. Wasps assigned to the social isolation and nonforaging groups were on the nest for a maximum of 48 h prior to being assigned a treatment group. Less than 48 h of experience is thought to have a small effect on influence performance, as wasps do not leave their nest to forage until they are 5 days old (Giray et al., 2005; Reeve, 1991; Shorter & Tibbetts, 2009). Furthermore, our previous work has shown that wasps need 5—7 days to accurately learn individual faces (Collard et al., 2024; Pardo-Sanchez & Tibbetts, 2022). Wasps remained in their treatment groups for 10—12 days before being brough back to the laboratory for testing. In 2023, we tested 31 wasps from nine nests, and in 2024, we tested 20 wasps from 11 nests.

### Rearing environments

Wasps were reared in one of three distinct environments: on the nest without the opportunity to travel long distances to collect food (nonforaging), on the nest with normal, long-distance foraging (foraging), or in social isolation (social isolation). In the nonforaging treatment, nests were enclosed with mesh on the bottom of the nestbox to prevent the wasps from leaving. Wasps in the nonforaging treatment were fed sugar, waxworms and water. Wasps collected this food by walking approximately 20 cm from their nest to the feeding station. Nests in the foraging treatment were left unaltered, allowing the wasps free access to forage naturally. *Polistes* travel for miles to collect nectar and caterpillars (Turillazzi & West-Eberhard, 1996), so long-distance foraging wasps gain extensive experience identifying floral resources, hunting caterpillars and navigating to and from their nest. We also provided ad libitum sugar, waxworms and water to wasps in the foraging rearing environment. To control for potential nest effects, we alternated which nests experienced each treatment condition. Wasps assigned to the isolation treatment were placed in 30.5 × 30.5 × 30.5 cm mesh containers, where they had access to sugar, waxworms, water and a small shelter. The social isolation containers were positioned in a field next to the nestboxes and covered by a shade cloth.

Unfortunately, methodological challenges prevented us from a full factorial experimental design with a nonsocial foraging treatment. When no other wasps are present on a nest, workers abandon their nest to hide. Wasps are extremely good at hiding, so we were unable to find and recapture solitary workers in large flight cages.

### Training and Testing

At 10—12 days old, we brought each wasp to the laboratory. We ensured consistency by housing all wasps in individual containers. The containers of wasps in the social treatment groups (foraging and nonforaging) were stacked so that they could see multiple wasps from their container. Previous work has shown that being housed within viewing distance of other wasps improves capacity for individual face learning relative to housing in complete social isolation (Pardo-Sanchez et al., 2022). Wasps in the social isolation treatment group were housed away from other wasps, so they did not have any visual social contact. After a rest period of 1—2 h upon arrival at the laboratory (day 0), we tested the wasps on a multi-access box task and an exploratory assay. The following day (day 1), we tested the wasps on colour learning and reversal learning (day 2). Wasps were rested on day 3, then tested on individual face learning on day 4. Wasps were typically not tested on the weekend, so there was variation in the length of time wasps were in the laboratory prior to each test. Total testing time was between 4 and 7 days. Cognitive performance was not influenced by the number of days a wasp was in the laboratory prior to testing (Supplementary Methods and Results).

### Multiaccess box

We followed the methods described by Corcoran and Tibbetts (2026). Briefly, each wasp was placed in a wooden box measuring 6.5 cm long, 5.5 cm wide and 1.5 cm tall (Supplementary Fig. S1). The ceiling of the box had three types of doors, each requiring a unique mechanism to open: push, pull or slide. Wasps were placed inside the box and given 10 min to escape using any of the doors. If a wasp did not escape within 10 min, we marked the trial as a failure. After three failures, the wasp was considered unable to complete the task. If the wasp successfully escaped, we recorded the type of door used and the escape time. Once a wasp escaped through a specific door type three times, we taped that door shut to encourage the wasp to explore alternative escape methods. The multiaccess box task assesses the wasps’ ability to innovate new escape methods, to learn from attempts and to demonstrate behavioural flexibility when familiar doors are no longer accessible (Auersperg et al., 2011; Johnson-Ulrich et al., 2018; Thornton & Lukas, 2012).

### Exploratory

We followed the methods described by Jomaa et al. (2024). Briefly, we placed each wasp in a 29 × 16 × 4 cm Plexiglas arena with 10 built-in wooden chambers (Supplementary Fig. S2). Wasps entered through a small hole that was subsequently covered with more Plexiglas. Exploration was measured as the number of unique individual chambers entered. Exploratory behaviour is highly repeatable within *P. fuscatus* (Jomaa et al., 2024).

### Colour learning and reversal

We followed similar methods to those described in Pardo-Sanchez et al. (2025). For the initial colour learning test, wasps were trained to associate a mild shock with one colour and no shock with a different colour (Supplementary Fig. S3). During training, each wasp was placed in a 2.5 × 4 × 0.7 cm box lined with stimuli of one colour (blue or yellow). In half of the trials, wasps received a mild, nonlethal shock (~0.4 V) for 2 min while viewing the negative stimulus colour (CS+). This shock was delivered via an electrified pad made of antistatic conductive foam powered by two copper wires connected to a Variac transformer. In the other half of trials, wasps were placed in a separate box where they viewed a different colour without receiving a shock. Between each trial, wasps rested for 1 min in a dark container with access to water. Each wasp completed five trials in each box, for a total of 10 trials. After completing all trials, wasps had a 45 min rest period in a dark container with access to water.

To assess learning and memory, we conducted a test consisting of 10 trials. In each trial, a wasp was placed in a rectangular box measuring 3 × 10 × 0.7 cm. The box featured two stimuli on opposite sides: the neutral stimulus on one side and the negative stimulus on the other. The floor consisted of the same pad used for training, but the pad was not electrified. At the start of each trial, the box was divided with two partitions: one displaying the correct stimulus and the other the incorrect stimulus. After a brief period in the antechamber, the partitions were simultaneously removed, allowing the wasp to choose a side of the box. A choice was recorded when the wasp’s head and thorax were within 2.5 cm of either side of the box. Following each decision, the wasp was placed in a dark container with water for 1 min. To avoid side biases, the position of the neutral stimulus was presented in a pseudorandom order. Wasps that learn and remember the initial training rapidly approach the correct stimulus. The test does not reinforce learning since no reinforcement occurs during the test.

Twenty-four hours after the initial colour test, we reversed the colour associations during training, pairing the previously neutral stimulus with shock and the previously negative stimulus with no shock. We trained wasps on this association for two training trials as opposed to the initial five training trials. Following these two trials, the wasps rested again for 45 min before their learning was assessed again using the 10-trial test described above. In previous work on workers from this same population, we used a memory test in between the initial and reversal tests to ensure the wasps had learned the stimuli correctly on the initial test (Pardo-Sanchez et al., 2025). We found that wasps performed significantly better than chance 24 h after the initial test, indicating that wasps remember the initial colour learning for at least 24 h.

### Faces

Wasps were trained and tested to differentiate between two different *P. fuscatus* wasp face images using the same methods used for initial colour training except face pictures were used as stimuli instead of colours.

### Ethical Note

Individual face learning, initial colour learning and reversal colour learning involved a low-level electrical shock that is aversive to wasps, but not lethal. The voltage was maintained at a level that did not harm the wasps. At the end of this experiment, the wasps were housed in the laboratory to be used in other studies.

### Statistical Analysis

We analysed the data in R version 4.4.1 (R Core Team, 2021). Individual face learning, initial colour learning and reversal learning scores ranged from 0 to 10, while multiaccess box scores ranged from 0 to 3. We compared learning across the three rearing environments on the four learning assays using generalized linear mixed effects models from the glmmTMB R package (Brooks et al., 2017). The dependent variable in the models was the total number of correct choices, and the independent variable was the rearing environment (categorical: foraging, nonforaging, social isolation). Nest ID was included as a random effect for all models. We initially included year as a random effect, but it explained no variance, so we removed it from the models. We assessed model fit using the DHARMa R package, which indicated that the models for individual face learning, initial colour learning and colour reversal learning were best fit with a Gaussian distribution, and the model for the multiaccess box was best fit with a beta-binomial distribution (Hartig, 2024). We used a type II Wald chi-square test to create an analysis of deviance table using the ‘Anova’ function in the ‘car’ package (Fox & Weisberg, 2019) to extract *P* values from the models. We calculated post hoc tests using the ‘emmeans’ R package using a Bonferroni correction for multiple comparisons (Lenth, 2021).

We used binomial tests from the ‘stats’ package to assess whether the number of correct choices in each group differed from the 50:50 random expectation (R Core Team, 2021). Binomial tests provide *P* values with no test statistics. Binomial tests were not performed for the multiaccess box because there was no clear random expectation, unlike for initial colour, colour reversal and face learning.

We also compared how rearing environments differed on the exploratory assay using a general linear model with exploratory score as the dependent variable and rearing environment as the independent variable.

## RESULTS

Wasps from different rearing environments opened different numbers of doors in the multiaccess box task (chi-square test: χ^2^_2_ = 9.14, *P* = 0.010; Fig. 1). However, wasps from different rearing environments did not differ in individual face learning (chi-square test: χ^2^_2_ = 0.97, *P* = 0.62), initial colour learning (chi-square test: χ^2^_2_ = 1.40, *P* = 0.50) or colour reversal learning (chi-square test: χ^2^_2_ = 1.68, *P* = 0.43). As multiaccess box performance differed across rearing environments, we followed up the initial test with a post hoc analysis to test how the three different rearing environments influenced performance. Foraging wasps escaped from more doors of the multiaccess box than nonforaging wasps (estimated marginal means: *z* ratio = 2.93, *df* = 47, *P* = 0.010). Isolated wasps had intermediate performance, so they did not differ from foraging wasps (estimated marginal means: *z* ratio = 1.40, *df* = 47, *P* = 0.071) or nonforaging wasps (estimated marginal means: *z* ratio = −0.35, *df* = 47, *P* value = 1.00). Lastly, exploratory score did not differ between rearing groups (chi-square test: χ^2^_2_ = 3.59, *P* = 0.17).

We used a binomial analysis to test whether wasps chose the correct stimuli more often than expected due to random chance in the face learning, colour learning and colour reversal tasks. We split the data by test type and rearing environment and performed a binomial analysis within each group. The binomial analyses showed that wasps in all rearing environments learned the initial colour and reversal tasks, as they chose the correct stimuli more often than expected by chance (Table 1). Foraging and nonforaging wasps also performed above chance level on individual face learning, while isolated wasps performed at chance level, indicating that they did not learn to differentiate the face stimuli (Table 1).

## DISCUSSION

The results of this study demonstrate that social and long-distance foraging experiences during early adulthood influence cognitive domains differently. Foraging experience positively impacted performance on the multiaccess box task, as foraging wasps escaped from more doors than nonforaging wasps (Fig. 1). In contrast, social experience had no effect on cognitive performance. Wasps from all three treatment groups performed similarly (Fig. 1), despite previous work showing that social experience influences cognitive development in *P. fuscatus* (Collard et al., 2024; Pardo-Sanchez et al., 2022; Pardo-Sanchez & Tibbetts, 2022; Tibbetts et al., 2019). Notably, foraging experience only influenced performance on the multiaccess box, but not performance on individual face learning, initial colour learning or colour reversal learning, indicating that aspects of cognition develop independently.

Wasps with foraging experience scored higher on the multi-access box test than nonforaging wasps, suggesting that the development of behavioural flexibility and innovation may be tied to experiences while foraging. Foraging involves trial-and-error instrumental conditioning to identify and gain access to rich food sources, the physical motor skills to gain access to flowers and hunt caterpillars, and the flexibility to change behaviour when food availability changes (Snell-Rood et al., 2015). This is similar to the behaviours involved in multiaccess box performance; trial-and-error instrumental conditioning and physical motor skills are needed to open multiple doors, and flexibility and inhibitory control are needed to try different doors once a particular door is blocked (Daniels et al., 2019). Previous work using multiaccess boxes directly mimicked foraging by using food rewards for opening a door (Benson-Amram et al., 2016; Daniels et al., 2019; Stanton et al., 2024). We found that foraging influenced multi-access box performance when we used escape as the reward, suggesting that this task required similar cognitive abilities as foraging despite not using food as the reward. To our knowledge, no other study has directly linked foraging experience with multiaccess box performance, but our finding does align with previous work showing that foraging and innovation are linked (Ducatez et al., 2015; Snell-Rood et al., 2015). Ultimately, more work is needed to quantify what aspects of foraging influence cognition. However, the results of the present study illustrate that foraging has important effects on cognitive development.

Despite its impact on the multiaccess box, foraging experience did not influence performance on other aspects of cognitive performance, including initial colour learning, colour reversal and individual face learning. It is particularly interesting that foraging experience did not influence colour reversal, as colour reversal and multiaccess box performance both measure aspects of behavioural flexibility. Perhaps foraging influences behavioural flexibility in physical motor tasks but not in behavioural flexibility in other contexts. Previous research shows that foraging influences the development of learning, memory and behavioural flexibility in honeybees (Cabirol et al., 2017; 2018; Carr-Markell & Robinson, 2014), so we expected to observe some additional cognitive differences between foraging and nonforaging wasps. Foraging may have different effects in honeybees and *Polistes* wasps because of the differences in early-life environments. Honeybees spend their early lives in dark, enclosed nests, and the onset of foraging marks a major developmental change associated with visual, behavioural and neural changes (Cabirol et al., 2018; Farris et al., 2001). In contrast, wasps emerge onto open nests where they are immediately exposed to a broader range of visual and olfactory cues. The results of the present study indicate that on-nest sensory experience may be sufficient for wasps to develop visual learning capabilities without foraging. Consistent with this result, laboratory-reared *P. fuscatus* lack foraging experience but can learn wasp faces, colours and reverse colour learning (DesJardins & Tibbetts, 2018; Pardo-Sanchez et al., 2022). Therefore, foraging experience is not necessary for the development of visual learning abilities.

One of the surprising aspects of our results is that the cognitive performance of isolated wasps did not differ from socially raised foraging and nonforaging wasps. Our previous work in *P. fuscatus* has shown that wasps reared in social isolation in the laboratory lack the ability to learn and remember conspecific faces and perform significantly worse on faces than socially raised wasps (Collard et al., 2024; Pardo-Sanchez et al., 2022; Tibbetts et al., 2019). Consistent with previous work, isolated wasps were the only rearing environment unable to learn conspecific faces due to performing at chance level (Fig. 1). However, individual face learning did not differ between isolated wasps and wasps in the two social rearing environments.

This study may differ from our previous work because previous development experiments focused on laboratory-reared wasps whereas this study focused on field-reared wasps. There is a growing appreciation that cognitive performance likely differs between field and laboratory environments (Ashton et al., 2018; Cauchoix et al., 2017; McCune et al., 2019; Morand-Ferron et al., 2016). For example, field-reared skinks outperformed laboratory-reared skinks on a spatial learning task (Vardi et al., 2020). Our data suggest that two main factors contribute to the difference between this study and previous work in laboratory-raised wasps. First, social, field-reared workers may be less adept at individual face learning than social, laboratory-reared workers. Consistent with this explanation, laboratory-raised wasps reared and trained on conspecific faces at the same time as this study made a mean (± SD) of 7 ± 1.34 correct choices out of 10 (Pardo-Sanchez et al., 2025), while field-raised wasps from this study averaged 6 ± 1.45 correct choices out of 10. Stressors such as predation and extreme temperatures may reduce cognitive performance in the field (Cabirol et al., 2018; Cauchoix et al., 2020). Second, field-isolated workers may perform better than laboratory-isolated workers. Field-isolated wasps receive more stimulation over their entire lifetime than laboratory-isolated wasps, as the field provides more visual stimulation and a more variable environment. Furthermore, some field-isolated wasps had more social experience (up to 48 h) than laboratory-isolated wasps (up to 24 h), and this additional social experience prior to isolation may improve face learning performance. Consistent with improved performance in field-isolated wasps, field-isolated wasps averaged 5.79 ± 2.19 correct choices out of 10 in this study, while laboratory-isolated wasps tested at the same time as this experiment averaged 5.13 ± 1.63 correct choices out of 10 (Collard et al., 2024). These results highlight that apparently small differences in experience can influence performance, so it is important to measure cognitive development in a range of different environments.

Notably, isolation did not influence performance in any of the other cognitive tests (multiaccess box, initial colour learning, colour reversal). Previous work testing how isolation influences nonsocial cognition has mixed results, with studies finding negative, positive or neutral impacts on cognition (Cabirol et al., 2017; Dalesman & Lukowiak, 2011; Lambert & Guillette, 2021; Nettle et al., 2015; Riley et al., 2018; Schrijver et al., 2004). For instance, wolf spiders reared in isolation performed worse on a spatial learning test compared to socially reared individuals (Punzo & Ludwig, 2002). In contrast, isolated guinea pigs outperformed socially reared individuals on a spatial learning test, although guinea pigs are a highly social species (Machatschke et al., 2011). We initially expected isolated wasps to show impaired cognition because wasps are highly social and because previous work has shown that early-life isolation impairs social cognition and behaviour in social insects (Pardo-Sanchez & Tibbetts, 2022; Tibbetts et al., 2019; Z. Y. Wang et al., 2022). Isolation may not have influenced cognitive performance in the present study because the field-isolated wasps may have experienced a sufficiently enriched environment to show normal cognitive development. Such cognitive flexibility may be beneficial, as *Polistes* occasionally develop in social isolation when they enclose on a nest that was abandoned during their pupal period (Tibbetts et al., 2019). Perhaps isolation has different effects on cognition in different ecological contexts. Future work will be important to assess whether ecological context influences the effect of isolation on paper wasp cognitive development.

While many previous studies have tested cognitive development, few have assessed the combined effect of social and long-distance foraging experiences on cognitive development within the same study. Because previous work has shown that *P. fuscatus* have complex social interactions that influence behavioural development (Pardo-Sanchez et al., 2022; Tibbetts et al., 2019), we initially expected that isolation would have stronger effects on cognitive development than foraging experience. Instead, we found that long-distance foraging experience improved performance on the multiaccess puzzle box, while social experience had no effect on cognitive performance. The stronger effect of foraging experience is consistent with a recent metanalysis showing that physical enrichment typically has a greater effect on cognition than social enrichment (Lambert & Guillette, 2021). Most of the studies in the meta-analysis tested social and physical enrichment in different studies, so they did not directly compare the effects of different types of experience. Furthermore, many studies in the meta-analysis assessed model systems in the laboratory. Despite these differences in study design, it is interesting that physical experiences may often have stronger effects on cognitive development than social experiences, even in social species.

One of the novel aspects of this study is that we measured how multiple aspects of cognition are influenced by developmental environment during early adulthood, finding that experience influenced different aspects of cognition in different ways. Long-distance foraging experience impacted scores on one cognitive test, the multiaccess box, but not on the other three tests (individual face learning, initial colour learning and colour reversal). This suggests that the physical innovation and behavioural flexibility involved in solving the multiaccess box may develop independently from traits like learning and memory of visual stimuli. Furthermore, the mechanisms underlying performance on the multiaccess box may differ from those underlying performance on other tasks (e.g. neural systems, physiological mechanisms, genotype). This is consistent with previous work suggesting that social and ecological experiences differently impact brain development and cognitive performance (Barth et al., 1997; Brenes et al., 2016; Freas et al., 2012; Jernigan et al., 2021; Maleszka et al., 2009; Z. Y. Wang et al., 2022). Other work in guppies, *Poecilia reticulata*, focusing on how brain morphology correlates across cognitive domains found that optic tectum size correlated with learning performance while telencephalon size correlated with reversal learning performance (Triki et al., 2022). Measuring which types of tasks are more versus less linked across developmental context provides insight into both the development of cognitive structure and the mechanisms that underlie different aspects of cognitive performance. As a result, future research in additional taxa will be important to identify general patterns in how developmental environment influences different aspects of cognition.

Experience at any phase of development may influence cognitive performance. In the present study, we altered experience during early adulthood, while much previous work in vertebrates has tested experience prior to adulthood (Champagne & Curley, 2005). In vertebrates, early life experiences can have dramatic effects on behaviour (Curley & Champagne, 2016; Martin et al., 2015). For instance, barn owls have an early-life sensitive period for visual experience that impacts their ability to localize sounds as adults (Brainard & Knudsen, 1998). Early-life experience may have different effects in vertebrates and insects (Westwick & Rittschof, 2021), especially insects like wasps that undergo complete metamorphosis. Wasp larvae have very different lives than adults; they do not leave their nest cells, are completely dependent on adults for food and have only rudimentary vision. There is also extensive neural remodelling during insect metamorphosis, which may constrain the effect of larval experience on adult behaviour (Vea & Minakuchi, 2021). We still have much to learn about how the behavioural effects of experience differ across developmental stages as well as the parallels between insects and vertebrates.

In conclusion, this study illustrates that experience during early adulthood influences cognitive performance in a social insect, but the effects vary based on the specific environmental stimuli and the aspect of cognition being tested. Foraging experience influenced multiaccess box performance, but social experience did not influence performance on any cognitive task. Studying cognitive development in the field is time-consuming and methodologically challenging, so current data are limited. However, natural environments are composed of many interacting factors that may combine to influence development in new ways. Future research testing the effect of biologically relevant environmental variation in nonmodel organisms will help us develop a more nuanced understanding of cognitive development in real, complex environments.

## Supplementary Material

1

Supplementary Material

Supplementary material associated with this article is available at https://doi.org/10.1016/j.anbehav.2026.123561.

## Figures and Tables

**Figure 1.**
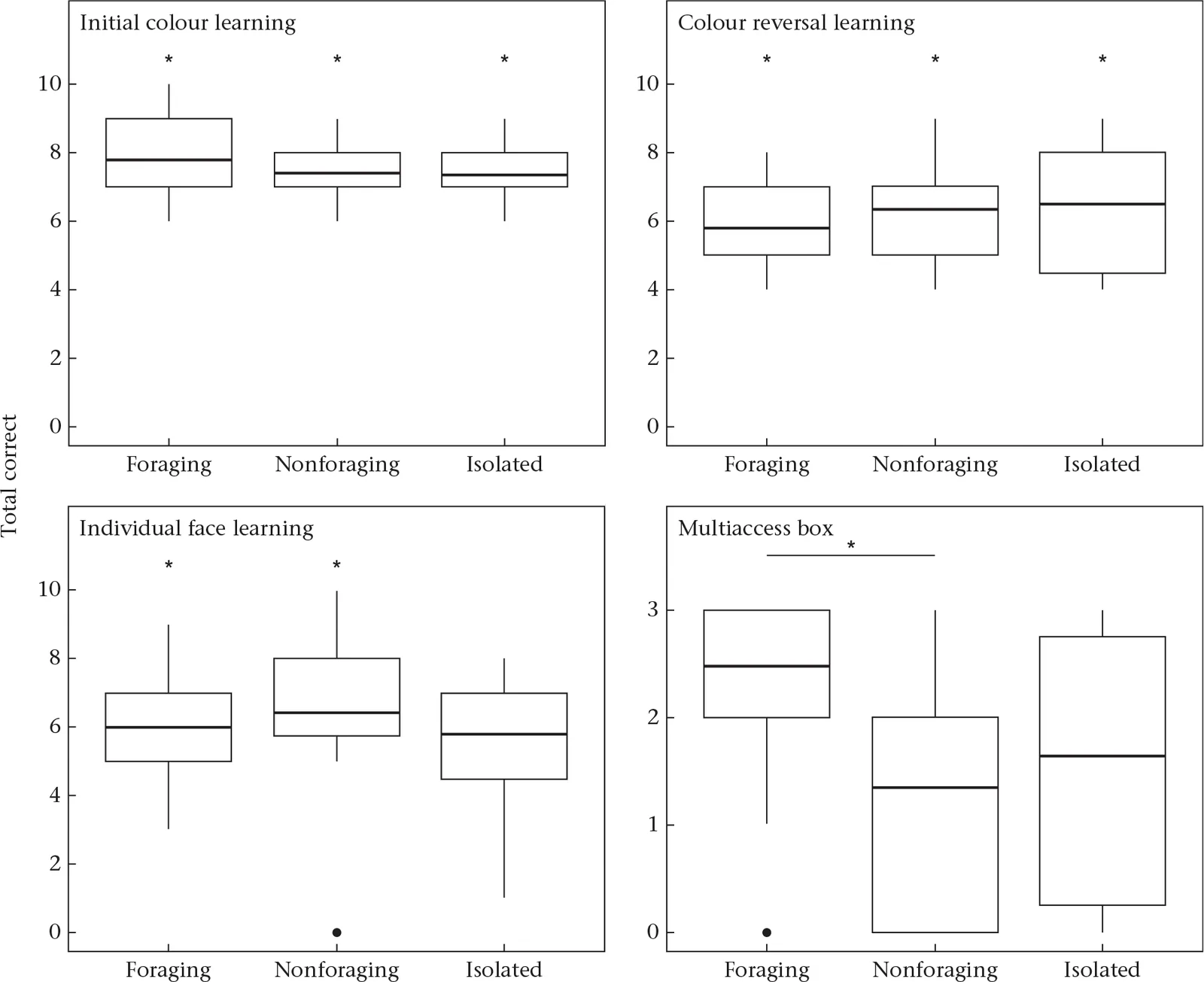
Scores across four different cognitive tests (colour learning, colour reversal, individual face learning, multiaccess box) for *P. fuscatus* raised in natural conditions (foraging), raised without being able to forage but with their nestmates (nonforaging) or raised in isolation. Box plots show the mean and first and third quartiles. Whiskers extend to the largest and smallest value no further than 1.5× interquartile range. *P < 0.05 (an asterisk directly above a box plot denotes that the group learned the stimuli, as they chose the correct stimuli more often that expect by chance; an asterisk centred between two groups denotes a significant difference between groups via post hoc testing).

**Table 1:** Results of binomial tests (*P* values) for each test type split by rearing environment

	Foraging	Nonforaging	Isolated

Individual face learning	**0.0057**	**<0.001**	0.076
Initial colour learning	**<0.001**	**<0.001**	**<0.001**
Reversal colour learning	**0.024**	**<0.001**	**<0.001**

Bold values indicate that wasps chose the correct stimuli significantly more often than expected by chance. No binomial tests were performed for the multiaccess box because there was no clear random expectation.

## Data Availability

The complete data set and R code used for analyses and plots are available as Supplementary Material.
