# Supplementary material for: The effects of social and foraging experiences across cognitive domains in Polistes fuscatus
Source: Anim Behav. Author manuscript; Available in PMC 2026 Nov 16. (PMC13771942; doi:10.1016/j.anbehav.2026.123561)
Supplement: Supplementary file 1 [file NIHMS2221026-supplement-1.zip › ANBEH-D-25-00587_SuppMat/SuppMat_Tibbetts.docx]

*Methods*

*Training and testing*

**Figure S1.** An image of the multiaccess box with a wasp escaping through the pull door. The multiaccess box had three types of doors through which wasps could escape, by pulling (left), sliding (centre) or pushing (right). Once a wasp escaped through a specific door type three times, we taped that door shut to encourage the wasp to explore alternative escape methods. If a wasp did not escape within 10 min, we marked the trial as a failure. After three failures, the wasp was considered unable to complete the task.

**Figure S2.** An image of the arena used to measure exploratory behaviour. Wasps were placed in the centre of the apparatus, which was made of wood and Plexiglas, and allowed to roam for 10 min. We measured exploratory behaviour as the number of unique chambers that wasps entered. Previous work has shown that exploratory behaviour is highly repeatable within *P. fuscatus* (Jomaa et al., 2024).

**Figure S3.** Schematic (adapted from Weise et al., 2022) of the training and testing arenas used for individual face learning. (A) Training: wasps were placed in a wooden box with a Plexiglas ceiling for 2 min while viewing an unknown wasp face and receiving a mild electric shock. After receiving a 1 min rest, wasps were placed in a different box while viewing a different unknown wasp face and receiving no electric shock. This cycle was repeated a total of five times. (B) Testing: wasps were tested in a wooden rectangular box with a Plexiglas ceiling. The shock-associated face image and the neutral face image were on two removable doors on the far walls of each respective side. Wasps were placed in the centre of the rectangle, then the doors were removed to allow the wasp to walk to either side. Once a wasp approached the choice region, we recorded its decision. We assessed learning in each wasp over 10 trials and switched stimulus locations in a pseudorandom order. Similar arenas were used for initial colour learning and colour reversal with colour stimuli instead of face images.

*Statistical analysis*

We analysed the data in R version 4.4.1 (R Core Team, 2021) for two supplemental analyses. The first supplemental analysis tested whether the amount of time a wasp was housed in the laboratory prior to cognitive testing influenced performance. All wasps were tested in the same order (multiaccess box, initial colour learning, reversal colour learning, individual face learning). However, there was some variation in testing day, primarily because wasps were not tested on the weekend. To assess whether this variation influenced performance, we tested whether the number of correct choices on the cognitive task (dependent variable) was influenced by the number of days the wasp was in the laboratory prior to testing (numerical) and treatment group (categorical: foraging, nonforaging, isolated), using general linear mixed effects models from the glmmTMB R package (Brooks et al., 2017). We ran separate analyses for each test type because days in the laboratory was strongly influenced by test type given that tests were always run in the same order. We used Gaussian distributions for individual face learning, initial colour learning and reversal colour learning, and verified model assumptions using the DHARMa R package (Hartig, 2024). We used a type II Wald chi-square test to create an analysis of deviance table using the ‘Anova’ function in the ‘car’ package (Fox & Weisberg, 2019). We did not include a model for the multiaccess box because there was no variation; this test was always performed on the day the wasp was brought back to the laboratory.

We also ran a second supplemental analysis to test whether performance changed over the course of the 10-trial test for individual face learning, initial colour learning and reversal colour learning. We used generalized linear mixed effects models from the glmmTMB R package with a binomial distribution (Brooks et al., 2017). We verified model assumptions using the DHARMa R package (Hartig, 2024). The dependent variable was trial outcome (1 for correct choices, 0 for incorrect choices), and the independent variable was trial number (numerical). We included wasp ID as a random effect. We used a type II Wald chi-square test to create an analysis of deviance table using the Anova function in the car R package (Fox & Weisberg, 2019).

*Results*

Initial colour learning was not influenced by time in the laboratory prior to testing (chi-square test: χ^2^_1_ = 0.46, *P* = 0.50) or treatment group (chi-square test: χ^2^_2_ = 1.51, *P* = 0.47). Reversal colour learning was not influenced by time in the laboratory prior to testing (chi-square test: χ^2^_1_ = 0.67, *P* = 0.41) or treatment group (chi-square test: χ^2^_2_ = 1.41, *P* = 0.49). Individual face learning was not influenced by time in the laboratory prior to testing (chi-square test: χ^2^_1_ = 1.79, *P* = 0.18) or treatment group (chi-square test: χ^2^_2_ = 2.01, *P* = 0.36).

Performance on 10-trial tests did not differ across the trials for any of the tests, including individual face learning (chi-square test: χ^2^_9_ = 6.63, *P* = 0.68), initial colour learning (chi-square test: χ^2^_9_ = 4.68, *P* = 0.86) or reversal colour learning (chi-square test: χ^2^_9_ = 4.3, *P* = 0.89).

**References**

Brooks, M. E., Kristensen, K., van Benthem, K. J., Magnusson, A., Berg, C. W., Nielsen, A., Skaug, H. J., Mächler, M., & Bolker, B. M. (2017). glmmTMB balances speed and flexibility among packages for zero-inflated generalized linear mixed modeling. *R Journal*, *9*(2), 378–400. https://doi.org/10.32614/RJ-2017-066

Fox, J., & Weisberg, S. (2019). *An R companion to applied regression* (3rd ed.). Sage.

Hartig, F. (2024). *DHARMa: Residual diagnostics for hierarchical (multi-level/mixed)* *regression models* (Version R package version 0.4.7) [Computer software]. https://CRAN.R-project.org/package=DHARMa

Jomaa, F. W., Laub, E. C., & Tibbetts, E. A. (2024). Behavioral syndromes in paper wasps: Links between social and non-social personality in *Polistes fuscatus*. *Current Zoology*, *70*(5), 659–667. https://doi.org/10.1093/cz/zoad054

R Core Team. (2021). *R: A language and environment for statistical computing*. R Foundation for Statistical Computing. https://www.r-project.org/

Weise, C., Ortiz, C. C., & Tibbetts, E. A. (2022). Paper wasps form abstract concept of ‘same and different’. *Proceedings of the Royal Society B: Biological Sciences*, *289*(1979), Article 20221156. https://doi.org/10.1098/rspb.2022.1156
